# Graphene-Assisted Thermal Interface Materials with a Satisfied Interface Contact Level Between the Matrix and Fillers

**DOI:** 10.1186/s11671-018-2704-1

**Published:** 2018-09-10

**Authors:** Bo Tang, Xufei Li, Weiqiu Huang, Haogang Yu, Xiang Ling

**Affiliations:** 1 Jiangsu Key Laboratory of Oil and Gas Storage and Transportation Technology, Changzhou University, Changzhou, 213016 China; 2 Jiangsu Key Laboratory of Process Enhancement and New Energy Equipment Technology, Nanjing University of Technology, Nanjing, 211816 China

**Keywords:** Thermal interface materials, Carboxyl, Graphene, Interface contact

## Abstract

Reduced graphene oxide (RGO) and three-dimensional graphene networks (3DGNs) are adopted to improve the performance of thermal interface materials (TIMs). Therein, the 3DGNs provide a fast transport network for phonons, while the RGO plays as a bridge to enhance the phonon transport ability at the interface between the filler and matrix. The types of surface functional groups of the RGO are found to exert a remarkable influence on the resulting thermal performance; the carboxyl groups are found in the optimal selection to promote the transport process at the interface area because a strong chemical bond will form between the graphene basal plane and epoxy resin (ER) through this kind of group. The resulting thermal conductivity reaches 6.7 Wm^−1^ K^−1^ after optimizing the mass fraction and morphology of the filler, which is 3250% higher than that of the pristine ER. Moreover, the mechanical properties of these as-prepared TIMs are also detected, and the specimens by using the RGO(OOH) filler display the better performances.

**Electronic supplementary material:**

The online version of this article (10.1186/s11671-018-2704-1) contains supplementary material, which is available to authorized users.

## Background

Thermal interface materials (TIMs) became one of hot issues during the last decade because of the increasing demands on dissipating heat of the highly integrated electron devices [1–4]. Compared with that of the traditional fillers (such as SiC, Al_2_O_3_, and BN), graphene displays a promising prospect to modify the epoxy resin (ER) based on its outstanding high thermal conductivity (5000 Wm^−1^ K^−1^ for the monolayer sample) [5]. Generally, the mass fraction of traditional fillers should excess 50% to satisfy the actual demand, leading to a poor mechanical performance of the resulting composites. On the contrary, a low ratio of the reduced graphene oxide (RGO) filler (~ 20 wt%) brings about a high thermal conductivity (~ 4 Wm^−1^ K^−1^) for the composite TIMs. Based on Balandin’s and Lu’s reports, the thermal conductivity enhancement factors reach ~ 2000% after adding the RGO modifier, and the observed mechanical properties meet the requirements for the practical application [6, 7]. Moreover, Chen et al. found that the graphene and carbon nanotubes can be used to further improve the thermal performance of the TIMs, simultaneously [8, 9].

However, the high defect density and poor continuity of the RGO (due to the violent oxidation-reduction reactions) limit the further enhancement of the resulting thermal performances [10]. Base on the report from Xie’s group, the phonon scattering mechanisms by vacancies in bulk materials and two-dimensional materials have been revealed [11]. For the two-dimensional RGO filler, missing mass and missing bonds caused by the defects impose a negative impact on the phonon transport. On the other hand, although the three-dimensional graphene networks (3DGNs) prepared by chemical vapor deposition method possess a high quality, the lack of an efficient link to achieve a favorable contact between the graphene basal plane and ER obstructs the phonon transport at their interface [12]. Recently, we found that a proper defect density of the 3DGNs is beneficial to the interface contact condition (plays the same role as the surface functional groups of the RGO), but the controlling process is quite complex [13]. Most recently, the RGO and 3DGNs were adopted as the co-modifier to improve the thermal performance of the TIMs by our group [14]. However, the resulting thermal performance is still far from expectation because the synergy between these two fillers is difficult to achieve.

In this study, the RGO fillers with optimized surface functional groups (including total amount and types) are fabricated and employed with the 3DGNs for the composite TIMs. Therein, the 3DGNs provide a fast transport network for phonon, while the RGO acts as the bridge to connect the graphene basal plane and ER. The influence from the types of the surface functional groups of the RGO is revealed, and a corresponding optimization design is carried out. The resulting thermal conductivity reaches 6.7 Wm^−1^ K^−1^ by adopting the optimized RGO filler, which is 25% higher than the previously reported graphene-based TIMs [7, 10]. Besides the influence on the thermal performances, the corresponding influences on the mechanical properties of the resulting TIMs from the functional groups of the RGO are also discussed.

## Results and Discussion

**Fig. 1:**
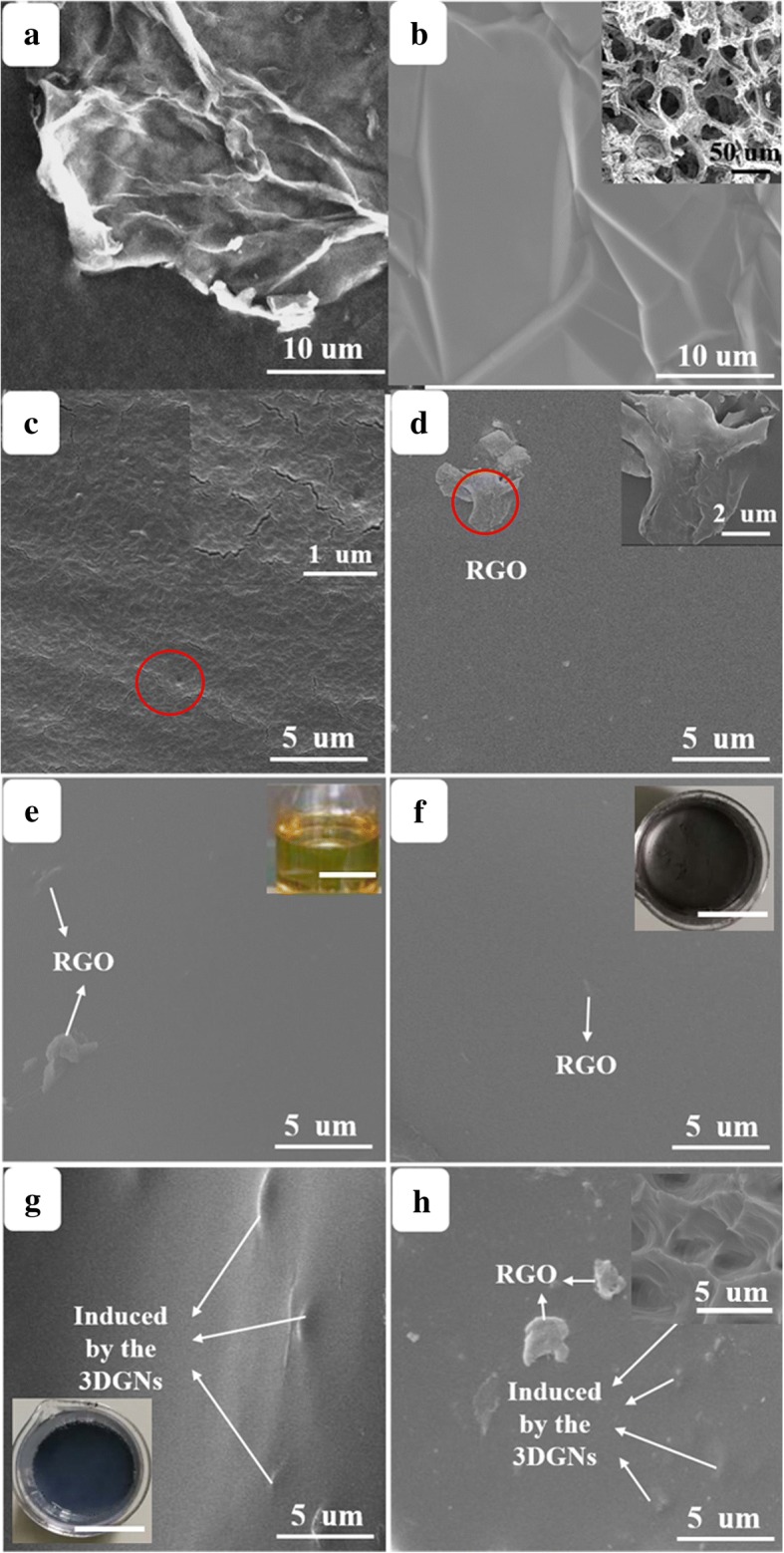
SEM images of the **a** RGO(OOH), **b** 3DGNs, **c** pristine ER, **d** RGO(OOH)-ER, **e** RGO(OH)-ER, **f** RGO(O)-ER, **g** 3DGNs-ER, and **h** 3DGNs-RGO(O)-ER. The digital photos of the ER, RGO filler, and RGO-3DGNs-ER are supplied in the insets of **e**–**g**, and all the scale bars represent 2 cm. The cross-sectional view of the SEM images is shown in the insets of **h**. SEM images of the pristine RGO, 3DGNs, and resulting TIMs are shown in figure, and the as-prepared composite TIMs display the smooth appearances (the digital photos of the ER, RGO filler, and RGO-3DGNs-ER are supplied in **e**–**g**). Different from that of the RGO, the size of wrinkles on the 3DGNs surface is much bigger (**a**, **b**). As for the RGO sample, the presence of wrinkles is spontaneous to enhance its stability, while the discrepancy between the thermal expansion coefficients of the graphene and nickel substrate leads to the wrinkles of the 3DGNs. A rough surface with obvious pores and cracks can be seen from the pristine ER, implying a poor thermal conductivity (**c**, the change of force constant resulting from the vacancies of the ER brings about a poor thermal conductivity) [11]. Contrarily, these cracks (forming during the solidification process) disappear after adding the graphene filler, which is in line with our previous reports [10, 12]. Moreover, partial RGO fillers can be seen on the surface of the RGO-ER specimens (**d**–**f**), while some obvious concave-convex (induced by the inner 3DGNs) appear on the surface of the 3DGNs-ER (**g**). Both these characteristics can be seen from the RGO and 3DGNs co-modified sample (**h**). The presence of the 3DGNs can be seen clearly from the cross-sectional view of the SEM images (insets of **h**)

SEM images of the pristine RGO, 3DGNs, and resulting TIMs are shown in Fig. 1, and the as-prepared composite TIMs display the smooth appearances (the digital photos of the ER, RGO filler, and RGO-3DGNs-ER are supplied in Fig. 1e–g). Different from that of the RGO, the size of wrinkles on the 3DGN surface is much bigger (Fig. 1a, b). As for the RGO sample, the presence of wrinkles is spontaneous to enhance its stability, while the discrepancy between the thermal expansion coefficients of the graphene and nickel substrate leads to the wrinkles of the 3DGNs. A rough surface with obvious pores and cracks can be seen from the pristine ER, implying a poor thermal conductivity (Fig. 1c, the change of force constant resulting from the vacancies of the ER brings about a poor thermal conductivity) [11]. Contrarily, these cracks (forming during the solidification process) disappear after adding the graphene filler, which is in line with our previous reports [10, 12]. Moreover, partial RGO fillers can be seen on the surface of the RGO-ER specimens (Fig. 1d–f), while some obvious concave-convex (induced by the inner 3DGNs) appear on the surface of the 3DGNs-ER (Fig. 1g). Both of these characteristics can be seen from the RGO and 3DGN co-modified sample (Fig. 1h). The presence of the 3DGNs can be seen clearly from the cross-section view of the SEM images (insets of Fig. 1h).

**Fig. 2:**
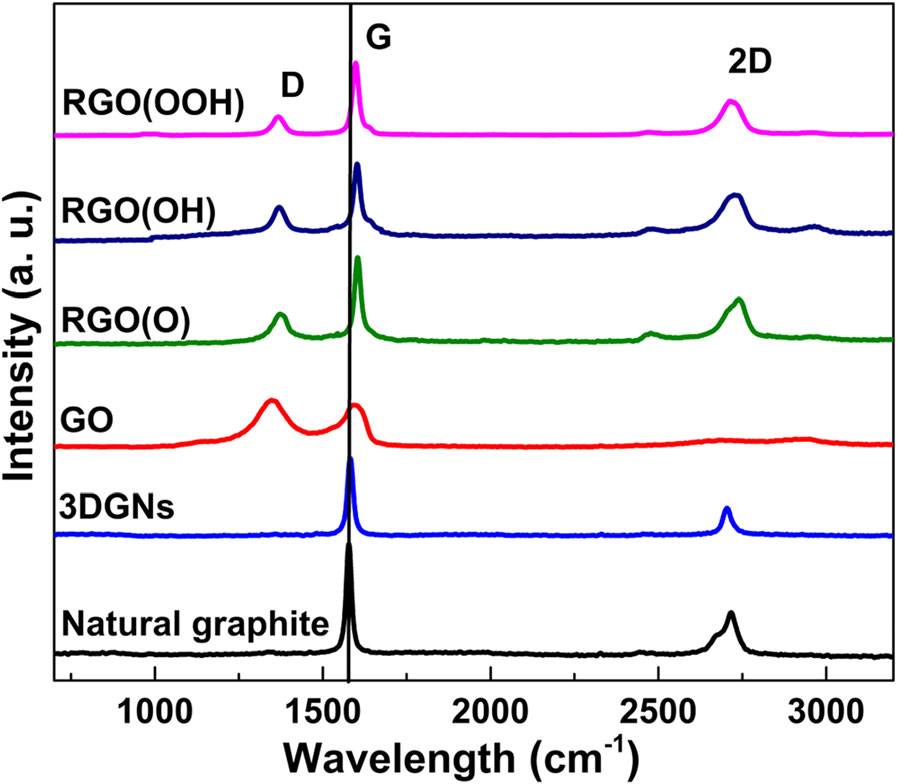
Raman curves of the natural graphite and various graphene fillers. The Raman curves of these employed RGO and 3DGNs specimens are recorded, and some remarkable distinctions on the relative intensities of the D, G, and 2D peaks can be found. The corresponding curve of the natural graphite is also recorded for comparison. The high quality of the 3DGNs is proven by the absence of the D peak in the corresponding curve, which is similar with that of the natural graphite. Contrarily, a remarkable D peak appears in the profile of the GO sample because of the introduced defects during the oxidation process. Moreover, the absence of the 2D peak confirms this point of view. After a reduction process, the intensity of the D peak decreases significantly and the 2D peak reappears in the curves of the RGO specimens. Based on the integral intensity ratio of the *I*_D_/*I*_G_, the defect densities of these adopted graphene samples can be calculated (all the results and detailed calculation are listed in Additional file 1: Table S1) [15, 16]. After analyzing these curves, it is found that the positions of the G band of the natural graphite and 3DGNs locate at 1580 cm^−1^, which shift to 1600 cm^−1^ for the RGO, confirming the higher quality of the 3DGNs compared to that of the RGO [17, 18]

In order to reveal the influence from the total amount and type of the surface functional groups of the RGO, various RGO fillers are used to modify the TIMs. The Raman curves of these employed RGO and 3DGN specimens are recorded (Fig. 2), and some remarkable distinctions on the relative intensities of the D, G, and 2D peaks can be found. The corresponding curve of the natural graphite is also recorded for comparison. The high quality of the 3DGNs is proven by the absence of the D peak in the corresponding curve, which is similar with that of the natural graphite. Contrarily, a remarkable D peak appears in the profile of the GO sample because of the introduced defects during the oxidation process. Moreover, the absence of the 2D peak confirms this point of view. After a reduction process, the intensity of the D peak decreases significantly and the 2D peak reappears in the curves of the RGO specimens. Based on the integral intensity ratio of the *I*_D_/*I*_G_, the defect densities of these adopted graphene samples can be calculated (all the results and detailed calculation are listed in Additional file 1: Table S1) [15, 16]. After analyzing these curves, it is found that the positions of the G band of the natural graphite and 3DGNs locate at 1580 cm^−1^, which shift to 1600 cm^−1^ for the RGO, confirming the higher quality of the 3DGNs compared to that of the RGO [17, 18]. In order to obtain more information of the surface functional groups of the RGO, XRD and XPS patterns are recorded and the corresponding types and ratios of various surface functional groups are calculated (Additional file 1: Figures S1, S2 and Table S2) [10, 12]. By adjusting the oxidation and reduction processes, the selective retention of various functional groups can be achieved (including carboxyl, hydroxyl, and epoxy groups) [19].

**Fig. 3:**
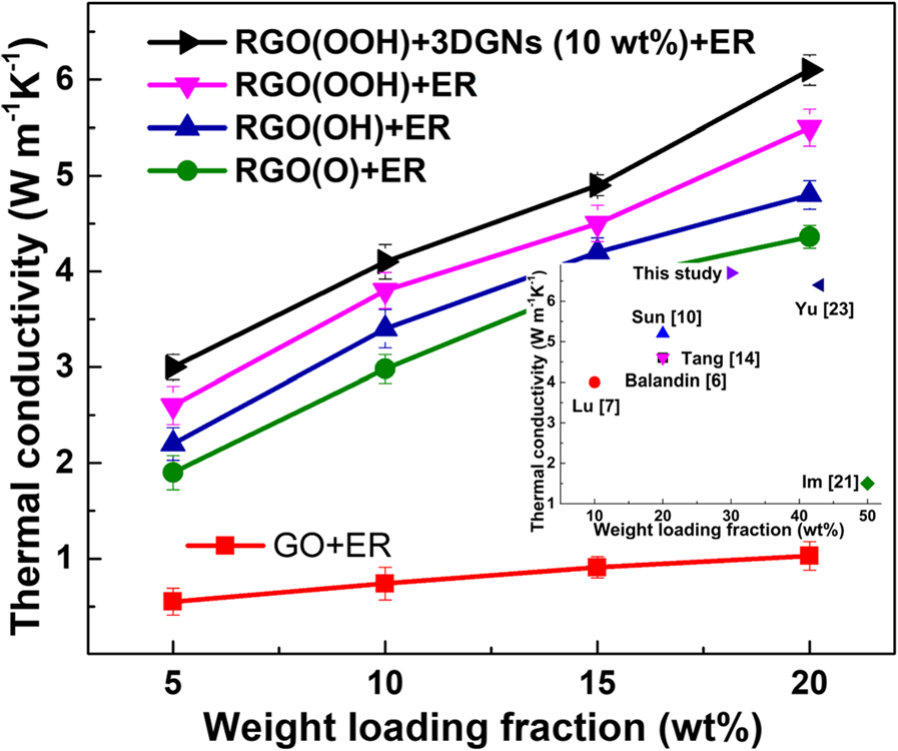
Thermal conductivities of various as-prepared composite TIMs with increasing mass fractions of the graphene fillers. The thermal conductivities of the resulting TIMs samples are shown in the figure, and the obtained thermal properties are closely related to the adopted RGO sample. Compared with those samples of adopting the RGO(OH) and RGO(O), the RGO(OOH)-assisted composite displays the better performances. The thermal conductivity (5.5 Wm^−1^ K^−1^) of the latter is about ~ 12% higher than that of the former (the mass fraction of the filler is 20 wt%), proving that the types of surface functional groups of the RGO exert a significant influence on the resulting thermal performance of the composite TIMs. Thermal conductivity of the as-prepared RGO(OOH)-3DGNs-ER is compared with that of the previous reported graphene-assisted ER (inset of the figure), implying adopting the RGO(OOH) is significant to achieve the high performance [6, 7, 10, 14, 20–23]. The thermal conductivity further increases after adding the 3DGNs (6.1 Wm^−1^ K^−1^), indicating adding the 3DGNs and a selective retention of functional groups of the RGO are both the determinants for the resulting thermal conductivities

The thermal conductivities of the resulting TIM samples are shown in Fig. 3, and the obtained thermal properties are closely related to the adopted RGO sample. Compared with those samples adopting the RGO(OH) and RGO(O), the RGO(OOH)-assisted composite displays the better performances. The thermal conductivity (5.5 Wm^−1^ K^−1^) of the latter is about ~ 12% higher than that of the former (the mass fraction of the filler is 20 wt%), proving that the types of surface functional groups of the RGO exert a significant influence on the resulting thermal performance of the composite TIMs. Thermal conductivity of the as-prepared RGO(OOH)-3DGNs-ER is compared with that of the previous reported graphene-assisted ER (inset of Fig. 3), implying adopting the RGO(OOH) is significant to achieve the high performance [6, 7, 10, 14, 20–23]. The thermal conductivity further increases after adding the 3DGNs (6.1 Wm^−1^ K^−1^), indicating adding the 3DGNs and a selective retention of functional groups of the RGO are both the determinants for the resulting thermal conductivities.

The interface boundary resistance (*δ*) is an important parameter to judge the interface contact condition. According to Balandin’s theory [24], the thermal conductivity of the graphene-modified TIMs can be calculated by the following equation:

1 $$ K={K}_g\left[\frac{2p\left({K}_g-{K}_e\right)+3{K}_e}{\left(3-p\right){K}_g+{K}_ep+\frac{\delta {K}_g{K}_ep}{H}}\right] $$

where *p* represents the volume percentage of the graphene filler and *K*, *K*_*g*_, and *K*_*e*_ are thermal conductivities of the resulting composite, graphene, and ER, respectively. *H* and *δ* are the thickness of the graphene and the thermal boundary resistance between the graphene and ER, respectively. Based on the relative calculations, it is found that the *δ* is deeply dependent on the specific surface functional groups of the adopted RGO (listed in Table 1), and the smallest value is obtained from the RGO(OOH)-assisted sample. These results are in line with the thermal conductivity results, confirming the types of functional groups of the RGO exert a significant influence on the interface contact level between the matrix and filler. As we know, the carboxyl group will react with the epoxy group under a middle temperature, and a chemical bond will form between the RGO(OOH) and ER during the solidification process (110 °C) [14, 25]. Moreover, the reduction degree of the RGO is closely related to the resulting thermal performances. Wang’s group had proven that the functional groups of graphene can reduce the phonon mismatch and enhance the thermal transport efficiency between the graphene basal plane and the ER in the theory [26]. Our group reported the relationship between the total amount of functional groups of the RGO and the resulting thermal conductivity of the RGO-ER [19]. Insufficient functional groups cannot provide an effective bridge to ameliorate the interface contact condition, while the function of excessive functional groups can be ignored because the total amount of phonon is limited. Recently, Manchado’s group and Araghi’s group reported similar influence from the functional group of the RGO on other organic composites [27, 28]. After optimizing the total amount of the surface functional groups (the ratio of element carbon atoms to functional carbon atoms in the RGO is *C*_element_:*C*_functional_ = 1.94:1), the thermal conductivity increases into 6.3 Wm^−1^ K^−1^.

**Table 1:** Thermal boundary resistances of these various samples

Fillers	3DGNs	RGO(O)	RGO(OH)	RGO(OOH)	RGO(OOH)-3DGNs
δ (× 10^−9^ m^2^KW^−1^)	6.3	4.4	3.8	2.5	2.8

**Fig. 4:**
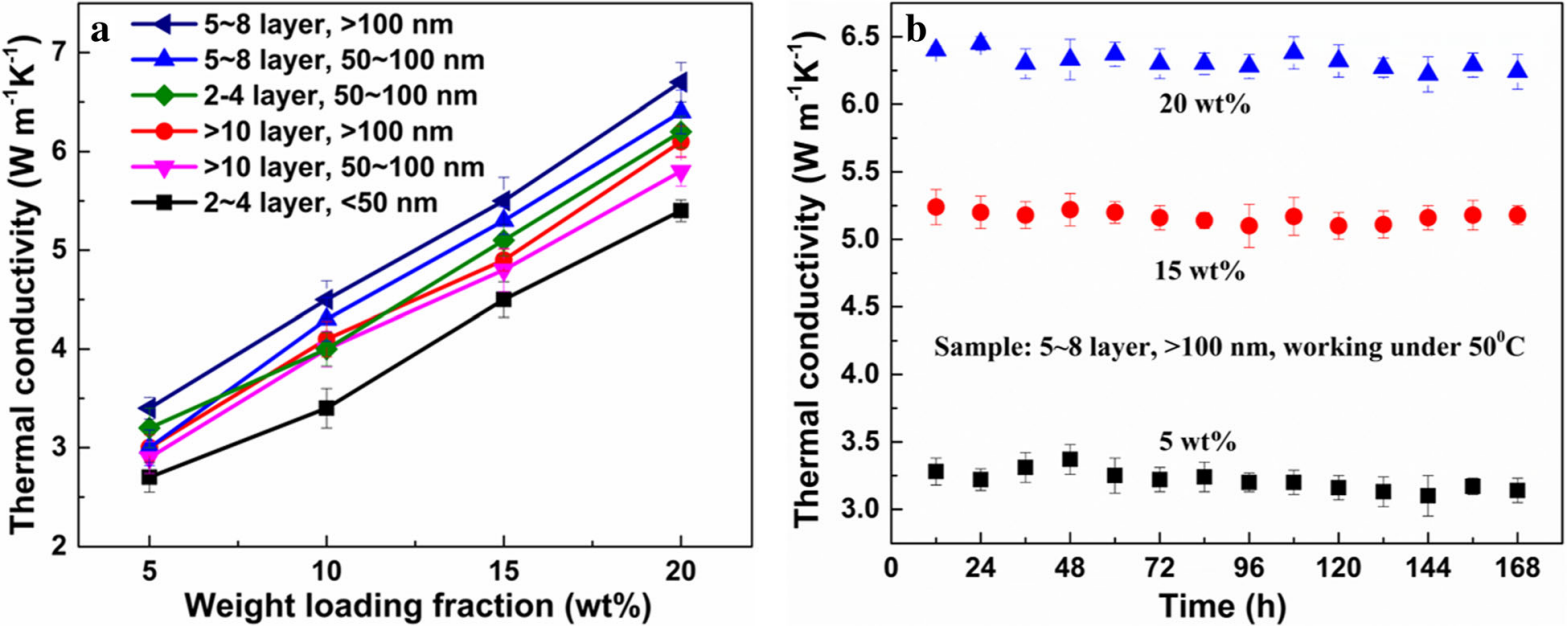
**a** Relationship between the thermal performances and the RGO morphology with increased mass fraction of the filler **b** thermal conductivity stability of the resulting TIMs with various mass fractions of the RGO filler under 50 °C for a long time. According to Balandin’s equation, the resulting thermal conductivity is also influenced from the morphology parameters of the graphene filler. Fu’s group optimized the morphology of the adopted RGO (nanoplatelets), which brings about a high thermal performance (4.01 Wm^−1^ K^−1^) [7]. Furthermore, our group discussed the detailed influence from the average size and thickness of the adopted RGO [10]. An average size (> 100 nm) and thickness (~ 2 nm) are recommended, and the thermal conductivity of the resulting TIM enhances to 6.7 Wm^−1^ K^−1^ (which is 25% higher than the previously reported values) [7, 10]. According to the obtained data (**a**), the influence on the resulting thermal conductivities from the average size of the RGO is more remarkable than the influence from the thickness of the filler, implying the contact area between the graphene basal plane and ER is the determinant for the obtained performance. Lastly, the mass proportions between the 3DGNs and RGO are optimized (10 wt% for the 3DGNs and 20 wt% for the RGO; although the thermal conductivity of the resulting TIMs almost increases linearly with the increased mass fraction of the graphene filler, a higher mass fraction of the filler will lead to a poor adhesiveness of the resulting TIMs) to achieve the synergy between them. A high stability of the thermal performances under a high temperature is vital to the TIMs to insure the electron devices working in the normal status. The thermal conductivities of the as-prepared TIMs with various mass fractions of the RGO(OOH) under 50 °C are listed in **b**, and no remarkable degradation can be seen after 7 days, indicating the promising prospect for the practical application

According to the Balandin’s equation, the resulting thermal conductivity is also influenced by the morphology parameters of the graphene filler. Fu’s group optimized the morphology of the adopted RGO (nanoplatelets), which brings about a high thermal performance (4.01 Wm^−1^ K^−1^) [7]. Furthermore, our group discussed the detailed influence from the average size and thickness of the adopted RGO [10]. An average size (> 100 nm) and thickness (~ 2 nm) are recommended, and the thermal conductivity of the resulting TIM enhances to 6.7 Wm^−1^ K^−1^ (which is 25% higher than the previously reported values) [7, 10]. According to the obtained data (Fig. 4a), the influence on the resulting thermal conductivities from the average size of the RGO is more remarkable than the influence from the thickness of the filler, implying the contact area between the graphene basal plane and ER is the determinant for the obtained performance. Lastly, the mass proportions between the 3DGNs and RGO are optimized (10 wt% for the 3DGNs and 20 wt% for the RGO; although the thermal conductivity of the resulting TIMs almost increases linearly with the increased mass fraction of the graphene filler, a higher mass fraction of the filler will lead to a poor adhesiveness of the resulting TIMs) to achieve the synergy between them. A high stability of the thermal performances under a high temperature is vital to the TIMs to insure the electron devices working in the normal status. The thermal conductivities of the as-prepared TIMs with various mass fractions of the RGO(OOH) under 50 °C are listed in Fig. 4b, and no remarkable degradation can be seen after 7 days, indicating the promising prospect for the practical application.

Beside the high thermal conductivity, a good mechanical performance is quite important to utilize the as-prepared TIMs in a large scale. The high intrinsic mechanical property of the graphene can be retained in the 3DGNs because of its relatively large size and continuous structure between the graphene sheets. The ultimate strengths (strain-stress relationship) and stretching limits of the pristine ER and the resulting TIMs are recorded (listed in Table 2; both the mass fractions of the adopted RGO and 3DGN fillers are 5 wt%). Based on the reports from Dermani’ group and Zhu’s group, the presence of surface functional groups of the RGO filler is closely related to the ultimate strength of the resulting TIMs [29, 30]. In this study, the RGO(OOH)-3DGNs-ER composite displays the best performances, indicating that the chemical contact between the RGO(OOH) and ER is stronger than that of other composites. The ultimate strength of the RGO(OOH)-assisted sample is ~ 10% higher than that of other TIMs. Similarly, its stretching limit reaches 280%, which is much better than that of the pristine ER. Therefore, the carboxyl groups on the RGO surface not only act as a bridge to promote the phonon transport between the filler and matrix, but also endow the TIMs a good mechanical performance because of the close chemical contact based on these functional groups. Moreover, the adhesiveness is another crucial property of the TIMs. The Young’s modulus and shear strengths of the pristine ER and the graphene-modified specimens are tested and listed in Table 3. As we can see, the corresponding performance of the 3DGNs-ER is inferior to that of the pristine ER due to the poor interface adhesive force between the 3DGNs and ER. Similarly, the performances of the RGO(O)- and RGO(OH)-assisted samples are not as good as that of the neat ER (because of the agglomeration of the RGO nanosheets), which is in line with the previous reports [31–33]. According to the study from Salom et al., a better joint strength can be achieved when a low mass fraction of the RGO filler is adopted to avoid the excessive agglomeration [33]. However, the low proportion of the graphene filler leads to poor thermal performances. On the contrary, the joint strength of the RGO(OOH)-3DGNs-ER is comparable with that of the neat ER, demonstrating the resulting adhesive strength is dependent on the functional group type of the adopted RGO filler. Based on the test results, the carboxyl group rather than the hydroxyl and epoxy groups imposes a positive effect on the mechanical and adhesive properties of the as-prepared TIMs. The RGO(OOH) filler plays the key role to ameliorate the interface contact level between the graphene basal plane and the ER.

**Table 2:** Mechanical performances of the as-prepared TIMs

Performances	Ultimate strength (%, relative to that parameter of the pristine ER)	Stretching limits (%)
Fillers		
None	100	220
3DGNs	104	260
RGO(O)-3DGNs	108	240
RGO(OH)-3DGNs	109	230
RGO(OOH)-3DGNs	121	280

**Table 3:** Young’s modulus and lap shear strength values of various TIMs at 22 °C

Performances	Young’s modulus (GPa)	Shear strength (MPa)
Fillers		
None	2.8 ± 0.1	9.7 ± 0.4
3DGNs	3.7 ± 0.2	7.3 ± 0.4
RGO(O)-3DGNs	3.8 ± 0.1	8.0 ± 0.3
RGO(OH)-3DGNs	3.7 ± 0.2	8.1 ± 0.5
RGO(OOH)-3DGNs	4.1 ± 0.2	9.4 ± 0.3

## Methods

### Materials

Natural graphite and acetone were received from Aladdin Co., Ltd. ER and curing agent were obtained commercially from Sanmu Co. Ltd. (Suzhou, China). Silver nitrate, potassium carbonate, ethanol, sodium hydroxide, phosphorus pentoxide, chloroacetic acid, hydrochloric acid, potassium permanganate, hydrazine peroxide, and sulfuric acid were purchased from the Beijing Chemical Reagent Plant (Beijing, China). Methyl ethyl ketone and sodium hydroxide were obtained from the Shanghai Chemical Reagent Co. Ltd. (Shanghai, China). Deionized water (resistivity 18 MΩ cm) was utilized to prepare all aqueous solutions.

### Preparation

The graphene oxide (GO) samples are prepared by the modified Hummer’s method and Zhang’s reported approach, and the major groups are carboxyl and hydroxyl, respectively [34, 35]. The major difference of Zhang’s approach compared with that of Hummer’s method is only one oxidation process is needed for the former. Briefly, 1.0 g natural graphite is added into 35 mL of H_2_SO_4_ (98 wt%), followed by the addition of 1.2 g KMnO_4_. The suspension is stirred for 72 h to fully engage H_2_SO_4_ intercalation. Then, 10.0 mL of deionized water is added and the temperature is heated up to 70 °C. Then, 10.0 mL of H_2_O_2_ (30 wt%) is introduced with a stirring process (5 h). Lastly, centrifugation and washing are performed to obtain the GO samples. Various reducing agents including alcohol and hydrazine are used to reduce the GO samples with selective functional groups. Briefly, 20 mg of GO sample is dispersed in 50 mL of ethylene glycol and a 60-min sonication treatment is performed. Then, the suspension is heated to 160 °C for 5 h under vigorous stirring. After a subsequent centrifugation process, the sample is washed by deionized water for three times. Lastly, the obtained paste is dried at 60 °C in a vacuum oven (both the carboxyl and hydroxyl groups are retained, while the epoxy groups are removed). As for using the hydrazine, all the functional groups are removed without selectivity. Briefly, 2 mL hydrazine is added into the 30-mL GO solution (2 mg mL^−1^) dropwise at 98 °C and kept for 4 h. Moreover, sodium hydroxide and chloroacetic acid are adopted to further control the RGO samples with designed functional groups [19, 24]. RGO(OOH): the natural graphite sample is prepared by the modified Hummer’s method and then reduced by the alcohol. RGO(OH): the natural graphite sample is prepared by Zhang’s method and then reduced by the alcohol. RGO(O): firstly, the natural graphite sample is prepared by the modified Hummer’s method. After that, the hydroxyl groups are transferred to the carboxyl group. Briefly, sodium hydroxide (1.2 g) and chloroacetic acid (1.0 g) are added into the RGO suspension (30 mL, 1 mg mL^−1^) and the mixture is bath-sonicated for 2 h. Lastly, the carboxyl groups of the intermediate product are removed by silver nitrate and potassium carbonate by Du et al.’s reported method [36]. Preparation of the TIMs has been described in our previous reports [14, 19]. In the first step, the RGO sample is dispersed in water (lysozyme is added and the pH value of the solution is adjusted to 10) [19] and is treated with ultrasonic for 10 min. Then, the well-dispersed RGO sample is poured into ER under modest stirring for 10 min. After stirring, the composite is cured at 110 °C for 2 h. The 3DGN sample is prepared by chemical vapor deposition method [13]. Briefly, nickel foam is heated to 1100 °C under Ar (300 sccm) and H_2_ (150 sccm) atmosphere with a 20 °C min^−1^ heating rate in a tube furnace to reduce the grain boundary of the substrate. Then, a small amount of CH_4_ (10 sccm) is introduced for 2 min. After that, samples are cooled down to room temperature under Ar (300 sccm) and H_2_ (200 sccm) atmosphere, and the cooling rates are 1 °C s^−1^, respectively. The preparation of 3DGN-modified samples has been described in our previous reports [10, 12, 14]. Briefly, a certain amount of 3DGNs is put into a mold, and then, the epoxy resin including curing agent is dropped on the 3DGN surface. After dropping a layer of epoxy resin (3DGN is covered), some 3DGNs are added again. Finally, the 3DGNs–epoxy resin mixture is cured at 110 °C for 5 h. The preparation of the 3DGNs and RGO co-modified composite is similar with that of the 3DGN-modified sample by replacing the pure ER with RGO-added ER (the mass fraction the RGO is 5–20 wt%). The average size of the RGO sample can be adjusted by adding a sonication treatment (0–12 h).

### Characterization

Morphology images were observed by the scanning electron microscope (SEM, FEI Sirion 200 working at 5 kV). Raman spectra were performed by the LabRam-1B Raman microspectrometer at 532 nm. X-ray photoelectron spectroscopy (XPS) profiles were recorded on a RBD upgraded PHI-5000C ESCA system. Laser flash analysis (LFA 2000, Linseis, Germany) and differential scanning calorimetry (Diamond DSC, PerkinElmer) were used to obtain the thermal performance of the composites. Thermal conductivities of the prepared composites are calculated by the following equation: *k* **=** *α* ∗ *ρ* **∗** *C*_*P*_ where the *k*, *α*, *ρ*, and *C*_*p*_ represent the thermal conductivity, thermal diffusion coefficient, density, and specific heat of the composites, respectively. The data of *α* and *C*_*p*_ can be detected directly from laser flash analysis and differential scanning calorimetry. Mechanical properties of these composites were recorded by a dynamic mechanical thermal analysis (DMTA, Triton Instrument, UK) instrument. The Young’s modulus was analyzed in dual cantilever bending mode using the DMTA (Triton Instrument, UK) instrument. Joint strength values of the prepared samples and pristine ER were abstracted by the single lap shear test by the ASTM D1002-01 standard with the DMTA (Triton Instrument, UK) instrument. Briefly, the aluminum pieces (100 × 25 × 2 mm^3^) were assembled into single lap shear joints with 12.5 mm of overlap length. The thickness of the TIMs was limited to 0.2 mm ± 0.04 mm, and the dimension of the overlapped joint was controlled to 25 × 12.5 mm^2^. Before the joint strength testing, a surface treatment process is performed to remove the dust and grease on the aluminum surfaces [33]. The aluminum pieces were treated by the abrasive blasting process, degreasing process (using methyl ethyl ketone), and etching process (by using NaOH solution (100 g L^−1^) at 60 °C for 5 min).

## Conclusions

The RGO and 3DGNs were adopted to modify the ER to improve the thermal performances of the resulting TIMs. By controlling the types of functional groups on the RGO surface, the corresponding influence on the interface contact level is revealed. Among all the as-prepared TIMs, the RGO(OOH) displays the best performance because of the high reaction activity of the carboxyl group (from the RGO) and epoxy group (from the ER) during the solidification process. Furthermore, the morphology (including average size and thickness) of the RGO filler is also adjusted to further enhance the thermal property. After the corresponding optimizing, the thermal conductivity of the resulting RGO(OOH)-3DGNs-ER reaches 6.7 Wm^−1^ K^−1^, which is 3250% higher than the pristine ER. Lastly, the mechanical properties and adhesiveness of these prepared specimens are tested, and the RGO(OOH)-added composites display the best performance because of the formed strong bond between the filler and matrix. Therefore, optimizing the type of the functional group of the RGO filler is a feasible way to enhance the thermal and mechanical properties of the composite TIMs.

## Additional file

Additional file 1: **Table S1.** The calculated defect densities of these graphene fillers based on the Raman patterns. **Table S2.** Ratios of carbon atoms from various chemical states in all the RGO samples based on XPS curves. **Figure S1.** XRD curves of the graphite, 3DGNs, and RGO. **Figure S2.** XPS curves of the (a) graphene oxide and (b) the RGO (OOH). (DOCX 996 kb)

## Abbreviations

3DGNs: Three-dimensional graphene networks
*C*_*p*_: Specific heat
DMTA: Dynamic mechanical thermal analysis
DSC: Differential scanning calorimetry
ER: Epoxy resin
GO: Graphene oxide
*k*: Thermal conductivity
RGO: Reduced graphene oxide
RGO(O): The RGO specimen with the epoxy as the primary functional group
RGO(O)-ER: RGO(O)-modified ER
RGO(OH): The RGO specimen with the hydroxyl as the primary functional group
RGO(OH)-ER: RGO(OH)-modified ER
RGO(OOH): The RGO specimen with the carboxyl as the primary functional group
RGO(OOH)-3DGNs-ER: RGO(OOH) and 3DGNs co-modified ER
RGO(OOH)-ER: RGO(OOH)-modified ER
RGO-3DGNs-ER: RGO and 3DGNs co-modified ER
sccm: Standard-state cubic centimeter per minute
SEM: Scanning electron microscope
TIMs: Thermal interface materials
XPS: X-ray photoelectron spectroscopy
*α*: Thermal diffusion coefficient
*ρ*: Density
