# Supplementary material for: Graphene-Assisted Thermal Interface Materials with a Satisfied Interface Contact Level Between the Matrix and Fillers
Source: Nanoscale Res Lett. 2018 Sep 10;13:276. doi: 10.1186/s11671-018-2704-1 (PMC6134478; doi:10.1186/s11671-018-2704-1)
Supplement: Supplementary file 1 — Table S1. The calculated defect densities of these graphene fillers based on the Raman patterns. Table S2. Ratios of carbon atoms from various chemical states in all the RGO samples based on XPS curves. Figure S1. XRD curves of the graphite, 3DGNs, and RGO. Figure S2. XPS curves of the (a) graphene oxide and (b) the RGO (OOH). (DOCX 996 kb) [file 11671_2018_2704_MOESM1_ESM.docx]

**Graphene assisted thermal interface materials with a satisfied interface contact level between the matrix and fillers**

The average size (nm) of graphene can be calculated by the integrated intensity ratio of I_G_/I_D_ from Raman curve according to the following equation [1, 2].

$L_{a}=43.5\times\frac{I_{G}}{I_{D}}$ (1)

Defect density can be roughly defined as (1/L_a_)^2^ in cm^-2^ [3-5]. Based on the ratio of I_G_/I_D_, the defect densities of various samples are calculated and listed in the Table S1.

**Table S1** The calculated defect densities of these graphene fillers based on the Raman patterns.

| Parameters  Samples | I_G_/I_D_ | Average size  (nm) | Defect density  (cm^-2^) |
| --- | --- | --- | --- |
| RGO (OOH) | 2.16 | 93.96 | 1.13×10^10^ |
| RGO (OH) | 1.93 | 83.96 | 1.42×10^10^ |
| RGO (O) | 1.69 | 73.52 | 1.85×10^10^ |

The morphology (including thickness and average size) of the RGO filler exerts a remarkable influence on the resulting performance of the TIMs. In our previous report, the specific impact has been discussed and revealed [8]. Average size of the RGO sheets can be adjusted by changing the centrifugal rate, and a higher centrifugal rate gives rise to smaller RGO sheets (rate range is from 4000 r/min to 7000 r/min). Average thickness of the RGO sheets is determined by the ultrasonic process, and the average number of RGO layers can be adjusted from >10 to 3 when the ultrasonic time increases from 15 min to 5 h. After comparing the resulting thermal performance of a series of samples with adopting various morphologies of the RGO fillers, both the larger average size and lesser layers of the RGO sheets bring about higher thermal performances because of the smaller total thermal boundary resistance from the interface area. In this study, the average size of the RGO is larger than 100 nm and the thickness is 5-8 layers (the centrifugal rate is 6000 r/min and the ultrasound time is 15 min). After using the as-prepared RGO filler, the obtained thermal conductivity reaches 6.1Wm^-1^K^-1^.

There are two fingerprint peaks located at 26^0^ and 44^0^ can be seen from the XRD curve of the graphite powder, graphene oxide and RGO (Figure S1, corresponding to (002) and (100) lattice planes, JCPDS card: 41-1487). The signal located at 44^0^ is induced by the long-term order of sp^2^ hybridized carbon atoms, which disappears in the curve of the RGO. Therefore, the 3DGNs sample fabricated by CVD method possesses a relatively high quality.


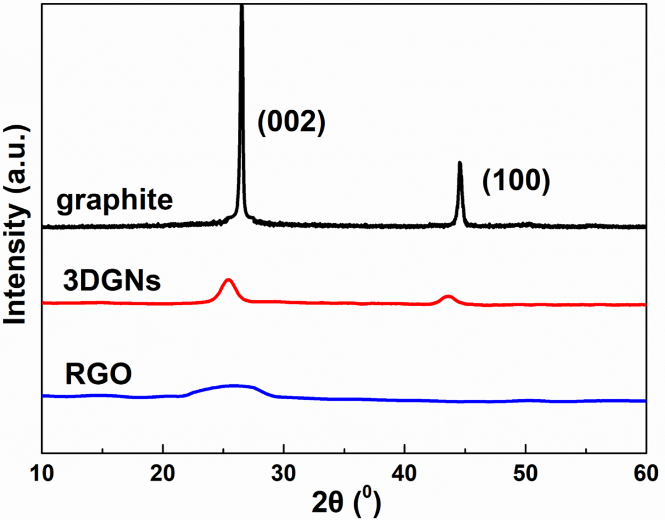


Figure S1 XRD curves of the graphite, 3DGNs and RGO.

Furthermore, the XPS patterns can be used to detect the chemical states of carbon atoms in the RGO. According to the studies from Zhang’s group, Massoud’s group and our group, the total amount and types of the functional groups on the surface of the RGO can be controlled by adjusting the reduction times and oxidizing (reducing) agencies [7-11]. The corresponding ratio of carbon atoms under various chemical states can be abstracted by the integrity intensity of these signals and have listed in the Table S2. The curves of the graphene oxide and RGO (OOH) are shown in the Figure S2.


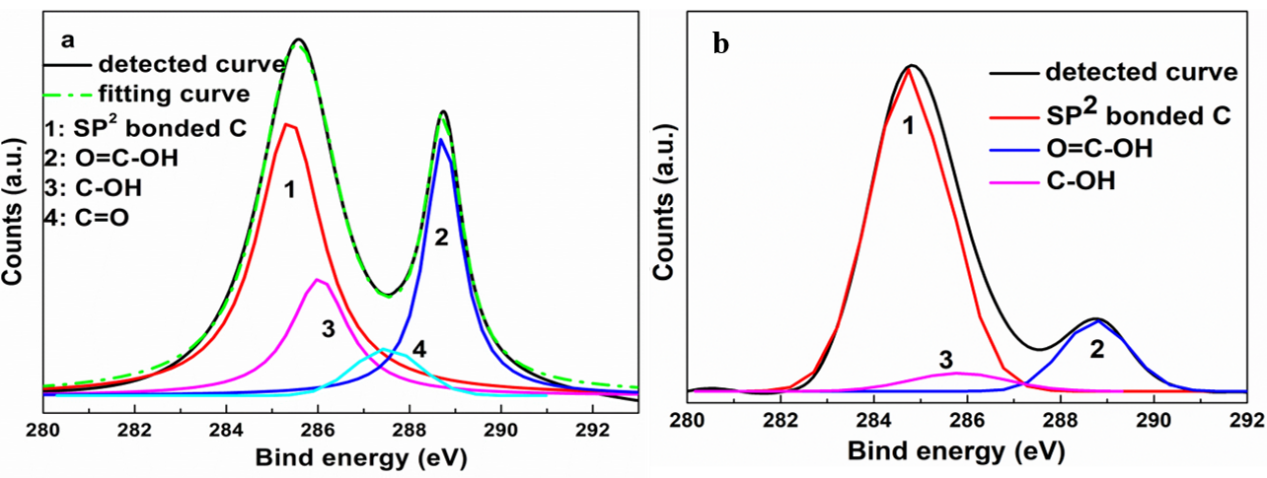


Figure S2 XPS curves of the (a) graphene oxide and (b) the RGO (OOH).

**Table S2** Ratios of carbon atoms from various chemical states in all the RGO samples based on XPS curves.

| Ratios  Samples | C_element_/C_functional_ | m_C_/m_O_ | C_element_:C_hydroxyl_:C_epoxy_:C_carboxyl_ |
| --- | --- | --- | --- |
| RGO (OOH) | 1.94:1 | 1.19:1 | 66:5:0:29 |
| RGO (OH) | 2.23:1 | 2.14:1 | 69:26:1:4 |
| RGO (O) | 1.86:1 | 1.70:1 | 65:2:24:9 |

**References**

[1] Tang B, Gao HY, Hu GX (2013) J. Phys. Chem. C. 117:25175-25184.

[2] Hu GX, Tang B, Min X (2012) Surf. Coat. Technol. 206:3586−3594.

[3] Chen JH, Cullen WG, Jang C, Fuhrer MS, Williams ED (2009) Phys. Rev. Lett. 102:236805-236809.

[4] Cancado LG, Takai K, Enoki T, Endo M, Kin YA, Mizusaki H, Jorio A, Coelho LN, Magahaes PR, Pimenta MA (2006) Appl. Phys. Lett. 88:163106-163108.

[5] Hwang JY, Kuo CC, Chen LC, Chen CH (2010) Nanotechnology 21:465705-465710.

[6] Sun YF, Tang B, Huang WQ, Wang SL, Wang ZW, Wang XB, Zhu YJ, Tao CB (2016) Appl. Therm. Eng. 103:892-900.

[7] Zhang GX, Xu YQ, Kuang K, Sun XM (2015) Sci China Mater 58:534–543.

[8] Xu C, Yuan RS, Wang X (2014) New Carbon Mater 29:61–66.

[9] White RL, White CM, Turgut H, Massoud A, Tian ZR (2018) J Taiwan Inst Chem E 85:18-28.

[10] Sun YF, He YF, Tang B, Tao CB, Ban JM, Jiang L (2017) RSC Adv 7:55790-55795.

[11] Hummers WS, Offeman RE (1958) J Am Chem Soc 80:1339.
